# Disseminating Adamantinoma of the Tibia

**DOI:** 10.1080/13577149778399

**Published:** 1997-06

**Authors:** Albert N. Van Geel, Hans M. Hazelbag, Rob Slingerland, Margit I. Vermeulen

**Affiliations:** 1 Department of Surgery Zuider-ziekenhuis Rotterdam The Netherlands; 2 Department of Surgical Oncology University Hospital Rotterdam/Dr Daniel den Hoed Cancer Center Groene Hilledijk 301 EA Rotterdam NL-3075 The Netherlands; 3 Department of Orthopaedic Surgery Academic Hospital Leiden The Netherlands; 4 Department of Chest Disease Zuider-ziekenhuis Rotterdam The Netherlands

## Abstract

*Patient*. This report describes a patient with a primary long bone adamantinoma. The lesion was initially wrongly diagnosed as fibrous dysplasia and the patient was treated by curettage. At second local recurrence, the tumour had progressed from an osteofibrous dysplasia-like to a full-blown classic adamantinoma, with metastatic potential to the lungs 19 years after the initial treatment. Lung metastasectomy by sternotomy was carried out twice in a period of over 3.5 years. The patient is currently alive without evidence of other metastatic disease.

*Discussion*. From the files of the Netherlands Committee on Bone Tumors, another five patients with lung metastaseswere studied. All types of adamantinoma should be treated by complete *en bloc* resection. For patients with metastatic spread to the lungs, close radiological follow-up and excision of tumour nodules seems to be the only logic treatment modality.


*Sarcoma* (1997) **1**, 109–111

CASE REPORT

### Disseminating adamantinoma of the tibia

ALBERT N. VAN GEEL,[1,2] HANS M. HAZELBAG,[3] ROB SLINGERLAND[4] & MARGIT I. VERMEULEN[1]

*Department of [1]Surgery and [4]Chest Disease, Zuider-ziekenhuis Rotterdam, [2]Department of Surgical Oncology, University Hospital Rotterdam/Dr Daniel den Hoed Cancer Center, Rotterdam & [3]Department of Orthopaedic Surgery, Academic Hospital Leiden, The Netherlands*

**Abstract**
*Patient.* This report describes a patient with a primary long bone adamantinoma. The lesion was initially wrongly diagnosed as fibrous dysplasia and the patient was treated by curettage. At second local recurrence, the tumour had progressed from an osteofibrous dysplasia-like to a full-blown classic adamantinoma, with metastatic potential to the lungs 19 years after the initial treatment. Lung metastasectomy by sternotomy was carried out twice in a period of over $3\frac{1}{2}$ years. The patient is currently alive without evidence of other metastatic disease.
*Discussion.* From the files of the Netherlands Committee on Bone Tumors, another five patients with lung metastases were studied. All types of adamantinoma should be treated by complete *en bloc* resection. For patients with metastatic spread to the lungs, close radiological follow-up and excision of tumour nodules seems to be the only logic treatment modality.

**Key words**: *tibia, adamantinoma, lung metastases, treatment.*

### Introduction

Adamantinoma of the long bones was first described by Fischer in 1913.[1] It was given its name because of the histological resemblance to the adamantinoma of the jaw (nowadays called ameloblastoma), but is regarded as a distinct clinicopathological entity.

Adamantinomas are rare skeletal neoplasms which account for about 0.3–0.5% of all malignant bone tumours. About 300 cases were reported up to 1994.[2–4] There is a slight predominance in males. The peak age incidence lies between 11 and 30 years, females being slightly younger than males. The definite site of predilection is the tibia, in which over 85% of adamantinomas occur. Within this bone, the tumour is usually found in the diaphysis, but it may also extend to the metaphysis. Occasionally, a multi-focal occurrence is present, in which tibia and fibula may both be affected. Rarely, a pretibial soft tissue location has been reported.[5,6]

The radiographic appearance varies from a small to extensive multi-lobulated radiolucent lesion in an area of bone destruction involving the anterior diaphysis. The cortex is mostly thinned and the periosteum may be elevated with a lamellar or solid periosteal reaction.[7–9] The radiological differential diagnosis includes fibrous dysplasia, (non-)ossifying fibroma or osteofibrous dysplasia (OFD), metastatic carcinoma and vascular sarcoma.

Histologically, the tumour consists of epithelial-like cells in strands or nests, embedded in a fibrous or osteofibrous tissue. Two main subtypes of adamantinoma are recognized: the so-called 'classic' adamantinoma with a predominance of epithelial tumour cells, and the 'OFD-like' or 'differentiated' adamantinoma in which the osteofibrous component dominates the lesion and inconspicuous epithelial elements can only be recognized after use of immunohistochemistry. Four basic patterns of epithelial differentiation—and combinations of these—may be encountered in classic adamantinoma: a basaloid pattern consisting of cell nests with peripheral pallisading, resembling basal cell carcinoma; a squamous pattern characterized by squamous cells with keratinization, keratohyalin granules and intercellular bridges, resembling squamous cell carcinoma; a spindle cell pattern with large areas of spindle cells, resembling fibrosarcoma; and a tubular pattern characterized by formation of channels, occasionally filled with erythrocytes and lined by cuboidal or flattened cells, resembling vascular tu-

Correspondence to: A. N. van Geel, Department of Surgical Oncology, Dr Daniel den Hoed Cancer Center, Groene Hilledijk 301, NL-3075 EA Rotterdam, The Netherlands. Tel: +31 10 4391506; Fax: +31 10 4864645; E-mail: geel@chih.azr.nl

1357-714X/97/020109-03 $9.00 ©1997 Carfax Publishing Ltd mours. Regardless of its histological variety, the epithelial nature of the tumour cells has been confirmed by immunohistochemistry and electron microscopy.[10–13]

The recommended treatment is wide *en bloc* resection. Amputation may not be avoidable when lesions contain a large soft tissue component or when local recurrences occur. The recurrence rate of patients treated just with biopsy or curettage is considerably higher.

Of particular interest are recent indications of a close relationship between adamantinoma and OFD, a benign lesion with similar clinicopathological characteristics. Opposite theories propose that adamantinoma may arise from OFD, or that OFD may result from reactive tissue overgrowing maturing neoplastic epithelial cells.[2,14–17] OFD-like adamantinomas in particular are sometimes difficult to distinguish from OFD, considering their clinical and histopathological features. This case report, which has previously been described in part (case 13)[2] shows the rarely reported progression of a primary OFD-like adamantinoma to a spindle cell classic adamantinoma at local recurrence, with eventual dissemination to the lungs.

### Case history

In 1974, a 13-year-old girl was seen because of a painful swelling of 1 month duration at the medial aspect of the diaphysis of the right tibia. At the age of six, she had twice sustained a fracture at this site. Conventional radiographs revealed a well-defined, multi-lobulated osteolytic lesion with intralesional opacifications and sclerotic margins in the anterior cortex. The lesion measured $8 \times 3 \times 2.5$ cm. The tumour was extensively curetted, leaving no macroscopic evidence of residual tumour; the site was then rinsed with carbolic acid and was filled with cancellous-bone chips. The histopathological diagnosis was fibrous dysplasia. Two-and-a-half years later the patient was treated in the same way for a recurrence. The pathological diagnosis was then changed to non-ossifying fibroma.

For a second recurrence with progressive symptoms, an *en bloc* resection was performed 5 years later. Routine pathological evaluation of the resected specimen revealed a large cyst bordered by numerous spindle-shaped cells. At the periphery of the tumour, strands of tumour cells infiltrated the cortex and the periosteum. Areas of loose fibrous tissue resembling the lesions identified in 1974 and 1977 were observed as well. Electron microscopic examination showed that the spindle-shaped cells contained microlumina, microvilli and mitochondria. There were several desmosomes between the spindle-shaped cells, a finding clearly characteristic of epithelial cells. On the outer site, the cell nests were bordered by basal lamina. Thus, the resection specimen contained an adamantinoma of the spindle cell subtype. Review of the original slides as well as immunohistochemical analysis of the primary tumour showed isolated and small aggregates of keratin-positive epithelial cells.

In 1993, more than $10\frac{1}{2}$ years after the last resection, two lung metastases were detected. There were no signs of other systemic spread and the two lesions were excised by sternotomy. The diagnosis of metastases of the adamantinoma, of the same spindle cell subtype, was confirmed by histology and immunohistochemistry. After $3\frac{1}{2}$ years, a new lung lesion was resected. The patient is now 22 years after diagnosis of the primary tumour.

### Discussion

The rate of distant metastases in long bone adamantinoma is about 15–20%.[4,8] This percentage may be higher because the indolent course of the adamantinoma necessitates long-term follow-up, and in the past, some patients dying from metastasizing adamantinoma may not have been diagnosed adequately. In their review in 1986, Moon and Mori found 14 metastases in 109 cases.[4] Distant metastases were mainly found in patients with a history of local recurrence, mostly due to inadequate initial treatment. Lungs are most frequently affected. Less frequently, metastases are found in regional lymph nodes, liver and bones. Pulmonary metastases appear as solid tumours of varying size and number in the lung parenchyma and the pleura. A very late appearance of the lung metastases, after 10 years or more, is not unknown.[18,19]

The histology of metastases of an adamantinoma shows the predominance of a spindle cell differentiation of the epithelial component, as in our patient. The osteofibrous component, which is of benign nature, is always absent. Because of the resemblance of this epithelial subtype to some spindle cell sarcomas like fibrosarcoma, the epithelial nature of the tumour cells can often only be confirmed after immunohistochemistry for keratins (the cytoskeletal proteins present in epithelial cells).

From the files of the registry of the Netherlands Committee on Bone Tumors containing approximately 10 500 patients registered between 1953 and 1996, 37 patients (0,35%) with an adamantinoma of long bone have been reported.[2,20,21] All of these cases were reviewed and the histopathological diagnosis was confirmed. In a follow-up study of 28 of these patients[2] with a mean duration of 10 years and 2 months, eight of these patients (29%) developed metastases, of which six were in the lungs. All of these patients had developed a local recurrence before the appearance of the lung metastases and four patients had regional or other distant metastases at the time of these lung metastases. Four of the six patients with lung metastases were treated by metastasectomy. Apart from our current patient, all eight patients with metastases have died of disease.

Their mean survival after diagnosis of the first metastasis was 4 years and 3 months; after diagnosis of the primary tumour, they survived 12 years and 8 months. The four deceased patients who underwent a lung metastasectomy had a mean survival of 4 years after metastasectomy. Our patient is alive 4 years after first metastasectomy without current evidence of metastases. However, in contrast to three of the four patients who died of metastatic disease, the lungs are the only location of detected dissemination in our patient until now.

Metastasectomy is the first-line treatment for pulmonary recurrences. In the Dutch series, radiotherapy and chemotherapy neither reduced the tumour volume nor improved survival.[2] To our knowledge, only one patient had at least stable disease for $4\frac{1}{2}$ years after treatment with several courses of various chemotherapeutical regimes and finally by radiotherapy (30 Gy/15 fractions).[22] With some selection based on prognostic factors such as disease-free interval, number of metastases and radical resection, a 5-year survival after metastasectomy of 36% is reported for a group of 5206 patients with a variety of malignant tumours and a 10-year survival of 26%.[23] Prognosis of metastases of an adamantinoma is probably better but data available in the literature are scarce. A rough approximation of the 5-year survival rate of disseminated adamantinoma appears to be in the range of 50–60%.[2,4,8]

### References

1 Fischer B. über ein primäres Adamantinom der Tibia. *Frankf Z Pathol* 1913; 12:422–41.

2 Hazelbag HM, Taminiau AHM, Fleuren GJ, *et al.* Adamantinoma of long bones. A clinicopathological study of thirty-two cases with emphasis on histological subtype, precursor lesion and biological behavior. *J Bone Joint Surg* 1994; 76A:1482–99.

3 Moon NF. Adamantinoma of the appendicular skeleton in children. *Int Orthop* 1994; 18:379–88.

4 Moon NF, Mori H. Adamantinoma of the appendicular skeleton—updated. *Clin Orthop* 1986; 204:215–37.

5 Bambirra EA, Nogueira AMMF, Miranda D. Adamantinoma of the soft tissue of the leg. *Arch Pathol Lab Med* 1983; 107:500–1.

6 Mills SE, Rosai J. Adamantinoma of the pretibial soft tissue. Clinicopathologic features, differential diagnosis, and possible relationship to intraosseous disease. *Am J Clin Pathol* 1985; 83:108–14.

7 Bloem JL, Van der Heul RO, Schuttevaer HM, *et al.* Fibrous dysplasia vs adamantinoma of the tibia. Differentiation based on discriminant analysis of clinical and plain film findings. *Am J Roentgenol* 1991; 156:1017–23.

8 Keeney GL, Unni KK, Beabout JW, *et al.* Adamantinoma of long bones. A clinicopathologic study of 85 cases. *Cancer* 1989; 64:730–7.

9 Mulder JD, Schütte HE, Kroon HM, *et al. Radiologic atlas of bone tumors.* Amsterdam: Elsevier, 1993; 255–7.

10 Knapp RH, Wick MR, Scheithauer BW, *et al.* Adamantinoma of bone. An electron microscopic and immunohistochemical study. *Virchows Arch (A)* 1982; 398:75–86.

11 Perez-Atayde AR, Kozakewich HPW, Vawter GF. Adamantinoma of the tibia. An ultrastructural and immunohistochemical study. *Cancer* 1985; 55:1015–23.

12 Rosai J. Adamantinoma of the tibia: electron microscopic evidence of its epithelial origin. *Am J Clin Pathol* 1969; 51:786–92.

13 Rosai J, Pinkus GS. Immunohistochemical demonstration of epithelial differentiation in adamantinoma of the tibia. *Am J Surg Pathol* 1982; 6:427–34.

14 Czerniak B, Rojas-Corona RR, Dorfman HD. Morphologic diversity of long bone adamantinoma. The concept of differentiated (regressing) adamantinoma and its relationship to osteofibrous dysplasia. *Cancer* 1989; 64:2319–34.

15 Hazelbag HM, Van den Broek LJCM, Fleuren GJ, *et al.* The distribution of extracellular matrix components in adamantinoma of long bones suggests fibrous-to-epithelial transformation. *Hum Pathol* 1997; 28:183–8.

16 Mirra JM. Adamantinoma and osteofibrous dysplasia. In: *Bone tumors. Clinical, radiologic, and pathologic correlations.* Philadelphia, London: Lea & Febiger, 1989; 1204–31.

17 Springfield DS, Rosenberg AE, Mankin HJ, *et al.* Relationship between osteofibrous dysplasia and adamantinoma. *Clin Orthop* 1994; 309:234–44.

18 Cohn BT, Brahms MA, Froimson AI. Metastasis of adamantinoma sixteen years after knee disarticulation. Report of a case. *Bone Joint Surg* 1986; 68:772–6.

19 Van Schoor JX, Vallaeys JH, Joos GF, *et al.* Adamantinoma of the tibia with pulmonary metastases and hypercalcemia. *Chest* 1991; 100:279–81.

20 Hazelbag HM, Fleuren GJ, Cornelisse CJ, *et al.* DNA aberrations in the epithelial cell component of adamantinoma of long bones. *Am J Pathol* 1995; 147:1770–9.

21 Hazelbag HM, Wessels JW, Mollevangers P, *et al.* Cytogenetic analysis of adamantinoma of long bones. Further indications for a common histogenesis with osteofibrous dysplasia. *Cancer Genet Cytogenet* 1997; in press.

22 Lokich J. Metastatic adamantinoma of bone to lung. A case report of the natural history and the use of chemotherapy and radiation therapy. *Am J Clin Oncol* 1994; 17:157–9.

23 Pastorino U, Buyse M, Friedel G, *et al.* Long term results of lung metastectomy: prognostic analyses based on 5206 cases. *J Thorac Cardiovasc Surg* 1997; 113:37–49.
